# Prospects of Bioactive Compounds in Designing Functional Foods: Challenges and Solutions

**DOI:** 10.3390/foods15081291

**Published:** 2026-04-09

**Authors:** Hadeel Edkaidek, Divakar Dahiya, Poonam Singh Nigam

**Affiliations:** 1 Department of Food Processing, Faculty of Agriculture, Palestine Technical University-Al-Aroub Branch, Hebron P625, Palestine; hadeeledkaidek@hotmail.com; 2 Basingstoke and North Hampshire Hospital, Basingstoke RG24 9NA, UK; 3 Biomedical Sciences Research Institute, Ulster University, Coleraine BT52 1SA, UK

**Keywords:** functional food, bioactive compounds, microencapsulation, probiotics, phenolic compounds, post-digestion bioactivity

## Abstract

There is an increasing interest in functional food formulations with added bioactive compounds, such as vitamins, probiotics, polyphenols and bioactive peptides, specifically in dairy and plant-based foods, bakery, and beverages. However, their stability in the food system, release rates and biological activity after consumption/digestion play an important role in the effectiveness of functional foods. There are technical challenges in maintaining the stability and acceptability of added compounds in the formulation design of food items. A novel approach to delivering bioactive compounds in functional foods is their microencapsulation, where stability-sensitive compounds are protected against their degradation during processing and physiological digestion, with targeted release in the gastrointestinal tract (GIT) and elicited cellular responses. Microencapsulation of bioactive compounds has been proven to be beneficial in in vitro models for the stability, antioxidant and immunomodulatory action, and acceptability compared to free (non-capsulated) forms. This technology is worth considering relative to the protection of health benefits of compounds used in food products, with their necessary bioactivity after physiological digestion in GIT. This article reviews important bioactive compounds, challenges, and strategies in the development of functional foods to ensure the required stability for the bioavailability of added compounds.

## 1. Introduction

In the literature, the term “functional foods” has been used in various contexts, reflecting its evolving and multidisciplinary nature. Rather than being strictly defined, functional foods are generally described as foods that provide health benefits beyond basic nutrition due to the presence of physiologically active components [1]. Importantly, this concept encompasses a broad spectrum of food categories, including naturally functional foods (e.g., fruits and fermented products), fortified foods, and foods enhanced through technological processes. Therefore, fortified foods represent only one subgroup within the wider functional food classification and should not be considered synonymous with the concept as a whole. According to Temple [1], functional foods are consumed as part of the normal diet and are distinct from pharmaceuticals, yet they can contribute to disease risk reduction and overall health improvement. Similarly, Butnariu and Sarac [2] emphasized their role in supporting physiological functions and well-being. While certain traditional or ancient foods may inherently contain bioactive compounds, their classification as functional foods depends on the application of modern scientific criteria, including demonstrated physiological effects and evidence-based validation. The concept of functional foods was first formally introduced in Japan in the mid-1980s, marking the transition from traditional dietary practices to scientifically substantiated health-oriented food design.

The term “functional food” has since been formalized into a legislative category known as FOSHU (Foods for Specified Health Uses), highlighting foods that are officially recognized for their health benefits. However, many fermented foods have been known since ancient times in many cultures, which have traditionally been consumed for their health-promoting properties. The popularity of dietary supplement practice has increased in several countries; as a result, numerous companies are marketing attractive brands of supplements in different forms and varying compositions so that consumers can purchase these commercial products through the internet. Consequently, it is important to consider the safety alerts to prevent any adverse events associated with the intake of dietary supplements. Kobayashi et al. analyzed the safety alerts associated with dietary supplements [3].

Bioactive compounds are the key components of functional foods, which positively influence essential physiological functions and play a role in lowering the risk of chronic diseases such as atherosclerosis, hypertension, myocardial infarction, and diabetes. Zheng et al. comprehensively reviewed the effects of bioactive compounds and food nutrients on the gut microbiota [4]. Among natural sources, plants in the form of fresh fruits and vegetables represent the richest reservoir of health-promoting bioactive compounds. The herbal beverages such as green tea, which are particularly rich in polyphenols potent antioxidants, help prevent the excessive accumulation of free radicals in the body [3,5]. Although fruits, vegetables, and green tea are rich sources of bioactive compounds, they are not automatically classified as functional foods unless their health-promoting effects are scientifically validated under the functional food criteria [1].

Functional foods typically include products supplemented with vital nutrients such as vitamins, minerals, certain fatty acids and dietary fibers; some products are produced with the action of specific live bioactives, for instance, probiotic microorganisms, including approved species of bacteria and yeasts. Plant bioactive compounds have been increasingly acknowledged for their ability to decrease the risk of certain illnesses as well as for stimulating physiological functions besides nutrition. El-Saadony et al. [6] reviewed immunological and nutritional aspects of bioactive compounds for food application and benefits for human health. Examples of food that have been ascertained to have probable health-giving properties range from cruciferous vegetables, fish and fish oils, citrus fruits, carrots, garlic, dairy products, tomatoes, oats, and high-fiber diets. Essa et al. have reviewed functional foods and reported these to have a positive impact on health [7].

Even with this information, merely adding bioactive compounds to a food system is not sufficient for ensuring their physiological activity. In this context, specific classes of bioactive compounds, including polyphenols, vitamins, and certain lipophilic compounds, require particular consideration due to their susceptibility to degradation [5,6]. Definitely, for such compounds to be active towards improving human health, they must be effectively released from the consumed food into a bioactive form during their transit process in the gastrointestinal tract (GIT). Lippolis et al. have studied the importance of the bioaccessibility and bioavailability of diet polyphenols and their modulation of gut microbiota [8]. These compounds may undergo degradation due to environmental factors such as oxidation, heat, pH variations, and enzymatic activity within different segments of the GIT, which can limit their stability and biological effectiveness [9].

Considering the instability of bioactive compounds, such as live cells of probiotic microbial species, during formulation and in the digestive system, microencapsulation has emerged as one of the promising solutions to overcome such problems. Encapsulation of bioactive materials in safe and protective micro-agent carriers can improve their stability during food processing and digestion, control their release patterns, and affect their bioavailability in the body after digestion, in that way improving their functionality. Microencapsulation is particularly important for bioactive compounds that are sensitive to oxidation, heat, pH fluctuations, enzymatic degradation, or those characterized by low bioavailability and rapid degradation in the gastrointestinal tract [10]. Prospects of a variety of bioactive molecules isolated from natural sources, including edible herbs and culinary spices, have been examined for food fortification, which can be delivered using sustainable encapsulation techniques [10]. Therefore, this article aims to discuss major bioactive compounds in functional foods and the concept of microencapsulation to protect their activities.

## 2. Bioactive Compounds in Functional Foods

A wide range of bioactive compounds are present in functional foods, which are responsible for their potential health benefits to consumers [4]. These compounds, such as vitamins, probiotics, phenolics, and bioactive peptides, exhibit biological activities in a series of mechanisms related to metabolic control, inflammation modulation, and antioxidant defense [6]. Importantly, their biological role depends not only on their chemical structure but also on their interactions with the food matrix, transformation during digestion, and interaction with biological targets. Arshad et al. recently published a report on the therapeutic potential and technological innovations of those functional foods, which have been enriched with bioactive compounds [11]. The following sections describe the major categories of bioactive compounds and their biological roles in health promotion.

### 2.1. Phenolic Compounds

Phenolic compounds represent one of the most abundant and structurally diverse groups of bioactive constituents naturally occurring in plant-derived foods, including fruits, vegetables, cereals, and plant-based beverages. This broad category includes flavonoids, phenolic acids, stilbenes, and tannins, which have been widely associated with health-promoting properties, including antioxidant activity, on gut microbiota [4]. Han et al. have reported the biological significance of dietary polyphenols in the modulation of metabolic pathways and anti-inflammatory effects [5].

Beyond their inherent chemical structures, the biological efficacy of phenolic compounds is strongly affected by their interactions with the food matrix and the transformations they undergo during gastrointestinal digestion. This usually gives rise to metabolites with bioactivity and bioavailability profiles substantially different from those of their native parent compounds, consequently determining their overall physiological outcome [6,8]. Therefore, it is necessary to consider their functionality in the digestive system rather than in the composition of food items [12].

Inflammation is a well-recognized indicator of cardiovascular disease, age-related disorders, and metabolic disorders. Maleki et al. described the significance of flavonoids for their anti-inflammatory effects [13]. Various phenolic compounds have been demonstrated to be able to control inflammatory responses by inhibiting pro-inflammatory cytokine release, such as interleukin-6 (IL-6) and tumor necrosis factor-α (TNF-α), or preventing nuclear factor-κB (NF-κB) activation via regulating significant pathways [5,13]. Ávila-Gálvez et al. studied the anti-inflammatory effect of polyphenols in an inflammatory model of human colon cells using extracts of *Echinacea purpurea* L. plant. Interestingly, studies in cellular models of intestinal inflammation demonstrated that phenolic metabolites, produced from flavonoids in ingested foods, inhibited inflammatory markers and reduced cytokine secretion [14].

Metabolic regulation in glucose and lipid metabolic processes is also associated with metabolic disorders such as diabetes and dyslipidemia. Sharma et al. reported the application of nano-engineered flavonoids in chronic metabolic diseases [15]. Evidence has shown that certain classes of phenolics can enhance insulin sensitivity, inhibit key enzymes in carbohydrate digestion, and alter the expression of genes related to lipid metabolism. Pleiotropic biological effects of dietary phenolic compounds and their metabolites have been studied by Villegas-Aguilar on energy metabolism, inflammation and aging [16]. Many of these effects take place by means of colonic fermentation and gastrointestinal digestion of metabolites. This highlights the need to consider the bioactivity that is only realized post-digestion, when assessing the functional potency of compounds for food-based interventions [12].

However, the practical incorporation of phenolics into functional foods faces major stability challenges. Exposure to heat, oxygen, and light during processing and storage can lead to degradation, while interactions with other food components may limit bioavailability [6,8]. To overcome these constraints, technological strategies such as microencapsulation and hybrid polymer matrices are increasingly being used to protect phenolics, control their release and enhance bioaccessibility. These strategies are aimed at maintaining the post-digestion functional activity of metabolites to ensure that the designed functional foods deliver the intended health benefits. Microencapsulation has been found to be an attractive approach for protecting bioactive food compounds from chemical degradation during food processing and gastrointestinal digestion. The protective environment offered by microencapsulation could promote not only the stability and bioavailability of pharmaceuticals but also the retention of functional properties after metabolic conversion [10,11].

Despite their well-documented biological relevance, phenolic compounds present substantial technological challenges that critically limit their incorporation into stable functional food systems. These compounds are highly susceptible to oxidative degradation, particularly under exposure to light, heat, and oxygen during processing and storage, leading to structural modifications that can significantly reduce their antioxidant potential and overall bioactivity [6,8]. In addition, phenolics are prone to chemical instability in varying pH conditions, as well as enzymatic and non-enzymatic transformations during gastrointestinal digestion, which can alter their native structures and generate metabolites with distinct and sometimes unpredictable biological activities [6,8]. While these transformations may enhance certain bioactivities, they also introduce variability that complicates the standardization of functional food formulations and challenges the reproducibility of physiological outcomes [12]. 

From a technological perspective, the interaction of phenolics with proteins, lipids, and polysaccharides within the food matrix can further limit their bioaccessibility and controlled release, thereby reducing their effective concentration at the target site [6,8]. To address these constraints, encapsulation-based delivery systems, particularly microencapsulation, have been widely explored to protect phenolic structures from environmental stressors and to improve their stability and controlled release during digestion [10,11]. However, despite these advances, limitations remain regarding encapsulation efficiency, scalability, and the precise control of release kinetics, especially when translating laboratory approaches into industrial-scale applications [10,11]. Therefore, a critical challenge in functional food design lies not only in enhancing the biological activity of phenolic compounds but in ensuring their structural stability and functional integrity throughout processing, storage, and digestion, which remains a key bottleneck in their practical application [12].

### 2.2. Bioactive Peptides

Bioactive peptides occur naturally in many food products, such as dairy (milk, cheese, and yogurt), soy, cereals, and legumes. Amino acids in a peptide chain can be broken down into short sequences labeled as bioactive peptides by enzymatic hydrolysis, or digestion in the gastrointestinal tract. They work as immune system modifiers, blood pressure reducers, and antioxidants, as well as anti-diabetic molecules. Peighambardoust et al. have highlighted in their review the health-promoting, biological, and functional aspects of bioactive peptides, suitable for their applications in food [17]. Moreover, their biological activities depend on many factors, including digestive enzyme resistance, molecular weight, and amino acid sequence, indicating the importance of studying the bioactivity in functional food after digestion. Aguilar-Toalá et al. reported that the strategy of encapsulation of bioactive peptides to improve the stability and protect the nutraceutical bioactivity for their food applications [18].

Some bioactive peptides have antihypertensive and metabolic effects, such as angiotensin-converting enzyme (ACE)-inhibiting properties. These have been found to play important roles in blood pressure regulation through their interaction with the renin–angiotensin system [18]. Other peptides can also affect glucose metabolism, either by improving insulin sensitivity or by inhibiting enzymes that break down carbohydrates, playing an important role in glucose control. Yu et al. recently published a report on the progress of research on bioactive peptides treating patients of Type II diabetes [19]. The ability of these peptides to withstand gastric digestion and be absorbed into the bloodstream has been shown to play a pivotal role in influencing their glucose-lowering activity. Chelliah et al. have also reported that bioactive peptides have a role in the control of diabetes and obesity [20].

Some bioactive peptides have antioxidant activity, which involves the scavenging of reactive oxygen species. Manzanares et al. have reported health-promoting effects of food-derived bioactive peptides, which can be improved using the strategies of their rational design and oral delivery [21]. Furthermore, many peptides show immunomodulatory activities; as a result, the gut barrier function is strengthened and the production of cytokines is regulated. Qiao et al. stated a definite role of dietary bioactive peptides in redox balance and metabolic disorders [22]. Such activities are especially beneficial for maintaining intestinal health and in the prevention of chronic inflammatory disorders [21,22].

Despite their promising health benefits, bioactive peptides face significant challenges related to stability and bioavailability during food processing and gastrointestinal digestion. Peptides are susceptible to enzymatic hydrolysis in the stomach and small intestine, which can alter their functional properties [17]. Their molecular weight, amino acid sequence, and resistance to digestive enzymes critically determine whether they retain their biological activity post-digestion [18]. 

The technological interventions are particularly important for peptides with antihypertensive and metabolic effects, as their efficacy depends on intact absorption and interaction with physiological targets, such as the renin–angiotensin system [21]. Furthermore, the diversity of peptide activities necessitates a tailored approach for their incorporation into functional foods. Some peptides exhibit glucose-lowering effects by improving insulin sensitivity or inhibiting carbohydrate-digesting enzymes, which are valuable for managing Type II diabetes [19]. Others contribute to antioxidant defense by scavenging reactive oxygen species and modulating endogenous antioxidant enzyme systems [21]. Additionally, immunomodulatory peptides can enhance gut barrier integrity and regulate cytokine production, supporting intestinal health [22]. These observations suggest that understanding the structure–function relationship and integrating rational design with advanced oral delivery strategies can maximize the physiological impact of bioactive peptides [21,22]. Such approaches highlight the need to move beyond simple dietary supplementation and toward functional food formulations that preserve peptide bioactivity during digestion and metabolic conversion [17,19].

Bioactive peptides represent a class of functional ingredients whose technological exploitation is inherently constrained by their structural fragility and susceptibility to gastrointestinal degradation, which directly affects their functional efficacy [17,18]. Unlike small-molecule bioactives, peptides are highly dependent on sequence-specific stability, where even minor alterations in amino acid composition can significantly modify their resistance to enzymatic hydrolysis and, consequently, their biological activity [17]. This structural sensitivity introduces a critical limitation in functional food applications, as peptides must retain their integrity not only during processing but also throughout digestion to exert systemic effects. Moreover, peptide–matrix interactions, particularly with dietary proteins and polysaccharides, may reduce bioaccessibility by limiting release from the food matrix, thereby lowering the fraction of peptides available for absorption [18].

From a technological standpoint, although encapsulation and delivery systems have been proposed to enhance peptide stability, these approaches face challenges related to low encapsulation efficiency, potential loss of bioactivity during processing, and inconsistent release profiles in the gastrointestinal environment [18,21]. Compared to more chemically stable compounds such as vitamins, peptides exhibit a narrower stability window, making their incorporation into functional foods more complex and less predictable. Furthermore, while some peptides demonstrate resistance to digestive enzymes, this property can also limit their bioavailability, creating a paradox between stability and absorption efficiency [21]. Therefore, the main challenge lies in achieving an optimal balance between structural protection and controlled release, which remains a major bottleneck in translating peptide bioactivity into consistent physiological outcomes in functional food systems [19,21].

### 2.3. Probiotics

Probiotics are defined as live microorganisms that, when administered in adequate amounts, confer a health benefit on the host [23]. They are found in some fermented foods such as yogurt, kefir, and some fermented products; however, not all fermented foods necessarily contain viable, well-characterized, and sufficient probiotic strains [23]. This distinction is critical, as many traditionally fermented foods rely on spontaneous fermentation processes, which do not guarantee the presence of clinically validated probiotic strains, in contrast to standardized probiotic formulations used in functional food development [1,23]. Unlike other bioactive compounds, probiotic microbial species act through their biological interactions that take place in the gastrointestinal tract. This makes their survival and functionality in GIT essential to determine their effectiveness in the functionality of food [24]. In contrast to non-living bioactive compounds such as polyphenols, probiotics exert their effects through dynamic host–microbe interactions, making their viability and metabolic activity key determinants of functionality [25].

The biological activities of probiotics are the result of their post-digestion survival and gut interaction, having the ability to withstand conditions of gastric juice, bile salts, and enzyme activities [25]. After consumption, probiotic cultures in functional food are capable of binding to the epithelial lining of the intestine, competing with other harmful microbes, as well as having the ability to modulate the intestinal microbiota [26,27]. This may not necessarily involve the survival of probiotic strains; however, their cells and metabolites in the form of postbiotics may still provide beneficial effects. This highlights an important functional continuum between probiotics and postbiotics, where even non-viable microbial components retain biological activity, thereby expanding the concept of bioactivity beyond viability alone [27,28]. Hijová examined the biotherapeutic potential of postbiotics, and therefore, the post-digestion functionality is also of significant consideration rather than just focusing on their survival [28].

The capacity of probiotic-rich food to regulate immune responses and lessen intestinal inflammation is among its most well-established benefits to health [29]. By controlling tight junction proteins, probiotics can strengthen intestinal barrier integrity, improve regulatory immune pathways, and affect cytokine production [30]. These effects are especially important for managing and preventing immune-related conditions, metabolic inflammation, and gastrointestinal disorders. Probiotics support metabolic health in addition to immune modulation by producing short-chain fatty acids (SCFAs), controlling lipid metabolism, and enhancing glucose homeostasis by restoring the diversity of the gut microbiome [31]. Some probiotic strains have also been linked to indirect antioxidant effects by either boosting host antioxidant defenses or lowering the oxidative stress via microbiota-mediated mechanisms, through an interplay between fermented food microbiota and gut microenvironment [32]. These results demonstrate probiotics as dynamic bioactive components in functional foods with systemic physiological effects to benefit human health.

In contrast, postbiotics are non-viable microbial cells, cell components, or metabolites that retain bioactive properties. Their efficacy does not rely on survival during gastrointestinal transit [28]. Postbiotics include short-chain fatty acids (SCFAs), peptides, and exopolysaccharides, which can interact directly with host cells and modulate gut microbiota composition [32]. These functional molecules generally exhibit a higher stability during food processing and storage, making them suitable for functional foods with extended shelf-life and consistent bioactivity [18]. Therefore, combining probiotics and postbiotics in functional food formulations may provide complementary health benefits, leveraging both live microbial activity and stable bioactive metabolites [28,32].

Despite the recognized benefits of probiotics, their efficacy in functional foods is often constrained by technological and physiological challenges. The survival of probiotic strains during food processing, storage, and gastrointestinal transit is highly variable and influenced by factors such as pH, temperature, osmotic stress, and enzymatic activity [25]. Strategies like microencapsulation, freeze-drying, and protective matrices have been shown to enhance probiotic viability, allowing a higher proportion of cells to reach the intestine and exert metabolic functions [24]. However, these approaches must be tailored for specific strains, as different species display variable resistance to environmental stressors and gastrointestinal conditions [26]. Consequently, the designing of functional food requires a strain-specific and delivery-oriented perspective rather than a uniform application of probiotics.

Moreover, the interplay between probiotics and postbiotics offers a promising avenue to overcome these limitations. While live probiotics provide dynamic interactions with the host gut microbiota, postbiotics comprising microbial metabolites, peptides, and exopolysaccharides can retain bioactivity independent of microbial survival [28]. This dual approach can stabilize functional properties during processing and storage while still delivering physiological benefits, including immunomodulation, antioxidant support, and metabolic regulation [32]. Integrating both probiotics and postbiotics into functional foods represents a rational strategy to maximize health benefits. This strategy highlights the importance of technological innovation, delivery systems, and understanding host-microbe interactions in the development of next-generation functional products [31,32].

In contrast to non-living bioactive compounds, probiotics introduce a distinct set of technological and biological challenges due to their reliance on cell viability as a prerequisite for functionality [23,25]. Their effectiveness is strongly dependent on maintaining adequate survival rates during processing, storage, and gastrointestinal transit, where exposure to acidic pH, bile salts, and enzymatic activity can lead to substantial loss of viability [25]. This intrinsic sensitivity positions probiotics as one of the most technologically demanding functional components, particularly when compared to structurally stable compounds such as vitamins or certain encapsulated peptides. Although protective strategies such as microencapsulation and freeze-drying have been developed to enhance survival, these approaches often result in heterogeneous protection across different strains, reflecting strain-specific variability in stress resistance [24,26].

Additionally, the requirement for high cell counts to achieve therapeutic effects further complicates formulation design, as maintaining sufficient viable populations throughout shelf-life remains a critical constraint [23]. From a mechanistic perspective, the functionality of probiotics is also influenced by their metabolic activity and interaction with the host microbiota, which introduces biological variability that is difficult to standardize across different individuals [24,25]. In comparison to postbiotics, which are inherently more stable due to their non-viable nature, probiotics offer dynamic biological effects but at the expense of reduced stability and predictability [28]. This trade-off highlights a key technological dilemma: while probiotics provide active host–microbe interactions, their incorporation into functional foods requires complex stabilization strategies that do not always guarantee consistent efficacy [27,28]. Therefore, future developments must focus on integrating strain-specific optimization with advanced delivery systems, while also considering complementary approaches such as postbiotic inclusion to overcome inherent stability limitations [32].

### 2.4. Vitamins

Vitamins are a diverse group of essential micronutrients required in trace amounts to maintain cellular homeostasis, support immune function, and regulate metabolic processes [33]. They occur naturally in various foods, including fruits, vegetables, and dairy products, yet food fortification has become a valuable strategy to enhance the nutritional value of functional foods. Traditionally, vitamins are classified according to their solubility: water-soluble (such as the B-complex group and vitamin C) and fat-soluble (vitamins A, D, E, and K). Tardy et al. have reported biochemical and clinical evidence on the role of vitamins for energy, fatigue and cognition [34].

Vitamin-related health benefits depend not only on their dietary content but also on their bioaccessibility during digestion and subsequent absorption into the bloodstream. Fat-soluble vitamins require efficient lipid digestion and micelle formation for uptake, while water-soluble vitamins generally diffuse more readily into the aqueous phase of the gastrointestinal contents [35]. Bioavailability of nutrients is further influenced by both intrinsic and extrinsic factors like food processing methods, the presence of dietary fats, and interactions, either synergistic or antagonistic, with other bioactive compounds [36]. Therefore, assessing vitamin functionality should focus on their fate after digestion and metabolic integration, rather than relying solely on food composition data.

Certain vitamins play key roles as essential components of the body’s natural antioxidant mechanisms. Indeed, vitamins C and E are effective scavengers for reactive oxygen species and prevent oxidative damage to cells [36]. B vitamins play a vital role as enzyme cofactors involved in energy production and signaling, while vitamins A and D are involved in regulating immunity and maintenance of epithelial integrity and barrier functions [37,38]. In essence, all these roles demonstrate that vitamins play a vital functional component, more than just basic nutritional functions. When their bioavailability and integration into body metabolic processes are appropriately taken into account, vitamins’ bioactivity is needed in the modulation of chronic disease risks and maintaining metabolic homeostasis [36,38].

Vitamins are even more prone to inactivation by food processing, storage, and their passage through the gastrointestinal tract [39]. In response to the instability and gastrointestinal delivery concerns, various approaches have been considered. These methods include optimizing the food vehicle and delivery systems based on the concept of encapsulation delivery strategies [40]. Such technological developments representatively demonstrate the function of employing a sophisticated delivery system in functional foods to attain the most effective biological activity after ingestion [41]. The stability and bioavailability of vitamins vary considerably depending on their chemical properties. Fat-soluble vitamins such as A, D, E, and K are highly sensitive to oxidative degradation and require lipid-based carriers or emulsification strategies to ensure proper absorption [40]. In contrast, water-soluble vitamins like C and B-complex are more susceptible to degradation by heat, pH variations, and enzymatic activity during gastrointestinal transit, which can significantly reduce their bioaccessibility [36,39].

To overcome these challenges, advanced technological approaches have been applied. Microencapsulation, nano-emulsions, and biopolymer-based coacervation techniques can protect vitamins from chemical degradation, mask undesirable interactions with the food matrix [10], and provide controlled release in the gastrointestinal tract [40]. For example, entrapment-based delivery systems have been shown to enhance the solubility and uptake of lipophilic vitamins, improving their functional efficacy after digestion [40,41]. Similarly, encapsulation of water-soluble vitamins in protein or polysaccharide matrices can shield them from enzymatic hydrolysis and pH-induced denaturation, thereby increasing bioavailability [10,11].

Overall, these findings emphasize that the functional potential of vitamins in fortified foods is not solely determined by their content but by the interplay between their chemical stability, food matrix interactions, and the delivery system employed. Optimization of these factors will ensure that vitamins retain their bioactivity for effective contribution to health benefits beyond basic nutrition [40,41].

Vitamins, although essential micronutrients, exhibit highly variable stability profiles that pose significant challenges for their incorporation into functional food systems [39]. Their susceptibility to oxidative, thermal, and photodegradation during processing and storage can lead to substantial losses in activity, particularly for water-soluble vitamins, which are more prone to degradation under environmental stress conditions [36,39]. In contrast, fat-soluble vitamins, while generally more stable, face limitations related to bioavailability, as their absorption depends on efficient lipid digestion and micelle formation, which can vary significantly depending on the food matrix and individual physiological conditions [35,40]. This duality highlights a key distinction: while some bioactives (e.g., phenolics) are primarily limited by chemical transformation, vitamins are constrained by both chemical instability and physiological dependency for absorption, making their functional outcomes more variable.

Technological strategies such as encapsulation, nano-emulsions, and biopolymer-based delivery systems have been proposed to address these challenges by improving stability and modulating release profiles [40,41]. However, these systems are not without limitations, as they may suffer from reduced scalability, potential interaction with food matrices, and incomplete release under physiological conditions, thereby affecting the consistency of vitamin delivery [40]. Moreover, the fortification of vitamins into functional foods does not always guarantee improved bioefficacy, as matrix effects and nutrient–nutrient interactions can either enhance or inhibit absorption [36]. Compared to probiotics, which require viability, and peptides, which depend on structural integrity, vitamins rely heavily on chemical stability and delivery efficiency, positioning them as a distinct class of bioactives with unique technological constraints. Therefore, optimizing vitamin functionality in food systems requires a comprehensive approach that integrates stability preservation, controlled delivery, and matrix compatibility, rather than relying solely on fortification strategies [40,41].

Table 1 presents a summary of bioactive components, their main food sources, and key physiological activities.

## 3. Functional Food

The growing evidence of the health benefits of bioactive compounds and their capacity to lower the risks of diseases has resulted in their increased fortification in functional foods. Rather than normally just adding compounds as loose ingredients in formulations of food, bioactive compounds can be incorporated within whole food matrices in a way that consumers can benefit from their physiological effects.

### 3.1. Dairy Products

Dairy products, due to their positive nutritional properties and universal consumer acceptance, act as ideal carriers for bioactive molecules. According to contemporary definitions, functional foods are foods that provide health benefits beyond basic nutrition due to the presence of bioactive compounds, and they may include naturally occurring or technologically enhanced products rather than being limited to fortified foods alone [1,2,7]. Probiotic microorganisms such as *Lactobacillus* and *Bifidobacterium* spp. have already established their applications in large-scale commercial production of yogurt, fermented milk, sour cream, kefir, and cheese, etc., benefiting consumers’ health by adjustment of microbial diversity in gut microbiota [24,29]. In this context, the interaction between diet and the gut microbiota plays a crucial role in host health, as nutrients and bioactive compounds can selectively stimulate beneficial microbial populations and metabolic pathways [4,32].

These dairy products offer an ideal microenvironment for probiotic cultures, ensuring that they receive the required nutrients, pH buffering, and protection from external environmental stress. For instance, commercial yogurt preparations fortified with *Bifidobacterium* spp., *Lactobacillus acidophilus*, and *Streptococcus thermophilus* have already stimulated functions in the GI tract and favorably influenced the intestinal balance [42]. Moreover, the efficacy of probiotics is closely linked to their ability to survive gastrointestinal conditions and exert beneficial effects on the intestinal barrier and immune responses [23,30]. Fermented-dairy-product-based formulations containing Bifidobacterium animalis subsp. lactis have demonstrated clinical efficacy in improving intestinal transit and reducing symptoms associated with functional bowel disorders, highlighting the functional role of probiotic microorganisms [11,27].

In addition to living bacteria, fermented dairy products are a good source of biologically active peptides produced by the hydrolysis of casein proteins in milk. These peptides, including Val-Pro-Pro (VPP) and Ile-Pro-Pro (IPP), which are produced from casein proteins, show angiotensin-converting enzyme (ACE) inhibition and have been found to decrease systolic blood pressure in mildly hypertensive subjects [43]. Bioactive peptides are increasingly recognized for their multifunctional biological activities, including antioxidant, anti-inflammatory, and metabolic regulatory effects, contributing to the prevention of chronic diseases such as diabetes and obesity [17,20,22]. Furthermore, fermented dairy products containing *Lacticaseibacillus paracasei* exhibit enhanced resistance to mucosal conditions and are associated with improved physiological responses in the gastrointestinal tract [27,44].

Through the fortification with bioactive compounds, dairy products are being investigated as a means of providing additional health benefits. It has been demonstrated that adding plant-derived phenolic extracts to yogurt, kefir, and cream cheese significantly increases their antioxidant and phenolic contents, enhancing their post-digestion bioactivity and potential health benefits for consumers [44]. Dietary polyphenols are known to exert pleiotropic biological effects, including modulation of oxidative stress, inflammation, and gut microbiota composition, although their bioaccessibility and bioavailability are influenced by digestion and metabolic processes [8,16]. Dairy product fortification with micronutrients like vitamin D is a practical way to improve population deficiency status and increase nutrient intake. Serum 25-hydroxyvitamin D concentrations are significantly higher in milk and yogurt fortified with vitamin D than in unfortified products, according to randomized controlled trials [45]. Moreover, micronutrients such as vitamins play essential roles in immune function, metabolism, and overall health, and their incorporation into functional foods enhances their nutritional and therapeutic value [33,38].

Encapsulation technologies play a crucial role in enhancing the functionality of dairy-based functional foods by protecting bioactive compounds and probiotic microorganisms from adverse processing conditions, including heat and pH fluctuations [10,18]. Techniques such as microencapsulation and immobilization within food matrices improve the thermal stability of sensitive compounds, ensuring their survival during processing and storage [18]. Furthermore, these systems enable targeted release in the gastrointestinal tract, thereby enhancing the bioaccessibility and bioavailability of bioactive peptides, probiotics, and micronutrients [9,10]. Consequently, the integration of encapsulation strategies within dairy products significantly strengthens their post-digestion bioactivity and overall functional performance [10].

### 3.2. Cereal-Based Products

Bakery products like fermented sourdough bread and cakes, as well as breakfast cereals, can be considered as versatile carriers of bioactive components. Functional foods, including cereal-based products, are defined as foods that provide health benefits beyond basic nutrition due to the presence of bioactive compounds or through technological enhancement strategies [1,2,7]. For example, bioactive peptides originating from milk and cereal proteins have been found to retain angiotensin-converting enzyme (ACE) inhibition activity after thermal processing and gastrointestinal digestion, which suggests potential application as a bioactive agent in systemic blood pressure regulation [46].

Moreover, bakery products can serve as efficient carriers of plant-derived bioactive compounds, particularly phenolic constituents. The incorporation of phenolic extracts or flours from different grains leads to increased total phenolic content, enhanced antioxidant activity, and improved technological properties such as rheology and texture. Dietary polyphenols are known to exert important biological effects, including modulation of oxidative stress and inflammation, although their bioaccessibility depends on gastrointestinal digestion and interaction with the gut microbiota [8,16]. A notable example is sourdough wheat bread produced using brewery spent grains carrying immobilized microbial cultures, which enhances fermentation efficiency and functional properties [47]. Additionally, fruit pomace has been utilized as a fortifying ingredient, resulting in elevated levels of polyphenols and improved radical scavenging activity in fortified bread products [48].

Furthermore, cereal-based dietary fibers act as prebiotic substrates that stimulate the growth and activity of beneficial gut microbiota, leading to the production of short-chain fatty acids (SCFAs), which are essential for maintaining intestinal health and metabolic homeostasis [4,27,49].

When probiotic lactic acid bacteria are added to bread, they partially survive after baking, enabling the delivery of their health benefits through solid food matrices [50,51,52]. Research has shown that cells from some strains, like *Saccharomyces boulardii* and *Lactobacillus acidophilus*, can survive baking [51]. Advanced delivery systems, including microencapsulation and immobilization techniques, have been shown to improve probiotic viability and stability during processing and storage, thereby enhancing their functional effectiveness [18,52]. Despite issues with processing conditions, reviews show that probiotics can be successfully added to processed foods like bread [52]. Essential vitamins and minerals have long been fortified in bread, according to systematic reviews; adding nutrients like folate (B9), vitamin C, and D to bread can increase their dietary intake for quantifiable health benefits [40,53].

Micronutrient fortification plays a critical role in addressing nutritional deficiencies, as vitamins are essential for immune function, energy metabolism, and overall health, while their bioavailability may vary depending on the food matrix and digestive processes [33,34,38]. Vitamin D3 and dietary fibers have been successfully added to white wheat bread in experimental studies, increasing the bread’s nutritional value without sacrificing its acceptable quality [40]. Cereal-based functional foods have been prepared excluding dairy materials to address the requirement of bioactive nutrition for the lactose-intolerant population [54]. In this context, non-dairy fermentation technologies enable the production of probiotic-rich cereal foods using alternative substrates, thereby expanding the availability of functional foods and supporting gut health through microbiome modulation [31,54].

Encapsulation strategies are equally important in cereal-based functional foods, particularly in improving the stability and delivery of heat-sensitive bioactive compounds during baking and processing [10,18]. The incorporation of encapsulated probiotics and phytochemicals within cereal matrices enhances their resistance to high temperatures and mechanical stress, thereby preserving their viability and functionality [51,52]. Additionally, encapsulation facilitates controlled and targeted release of bioactive compounds during gastrointestinal digestion, improving their interaction with the gut microbiota and increasing their bioaccessibility [4,9]. These mechanisms ultimately enhance post-digestion bioactivity, supporting the health-promoting potential of cereal-based functional foods [8,10].

### 3.3. Plant-Based Products

Functional beverages are excellent carriers for water-soluble bioactive ingredients. Wherever possible, the basic vitamins, C and B vitamins, and some phenolics have been added to fruit juices and plant extracts to improve their antioxidant and metabolic potency [55]. These compounds, however, easily get degraded during storage and digestion, and hence, there is a need to improve their formulation and stability [36,56]. Plant-based fermented matrices such as cabbage, soy, and cereals act naturally as probiotics and phenolics. Microbial fermentation makes bioactive compounds more bioavailable, hence contributing towards the maintenance of physiological balance in the body [57,58].

Bioactive peptides produced using enzymatic hydrolysis and/or microbial proteolysis also have positive health impacts. Biologically active peptides produced through microbial proteolysis in fermented soy beverages and cereals increase their antioxidant and signaling efficacy [17,59]. Peptides obtained from agro-industrial by-products such as cereal brans and soybean residual fractions have been demonstrated to possess numerous techno-functional properties useful in the development of high-quality food products [60]. Moreover, hydrolysates obtained from fruits and vegetables have been established to suppress oxidative stress and intestinal immune regulation [61]. Current knowledge demonstrates the potential usefulness of bioactive peptides in improving the nutritional and health properties of fermented food products. Sosalagere et al. reported functionalities of bioactive peptides isolated from fruits and vegetables [61].

## 4. Challenges in Functional Food Design

The challenge of developing functional foods is posed by a number of interrelated factors that range from market feasibility to technical and scientific credentials and from the consumers’ perspective, as health-related reservations continue to necessitate health literacy in science communication [62]. Making such bioactive compounds more affordable in terms of large-scale processing to ensure a broader market segment is also necessary. This must meet considerations of commercial viability and environmentally responsive processing [63]. Technically, while it is possible to introduce bioactive compounds successfully into food matrices, their stability during fortification becomes an issue. Bioactive compounds like vitamins and polyphenols tend to be unstable in environments of heat processing and pH variation. This tends to affect their biological activity significantly [36,64]. Another challenge is that while these substances have biological activities, their concentration above a certain point in foods might cause unwanted properties like bitterness and unacceptable colors. The non-standardized fortification will affect the acceptance of food by consumers [65].

With the increasing global demand for health-promoting foods, partly occasioned by the rising prevalence of chronic diseases and due to changing lifestyle patterns, the food industry for any fortification has to follow regulatory and safety frameworks for consumers’ confidence [66]. The need to address these complexities arises to translate laboratory-scale innovations into commercially viable and scientifically credible functional products. Table 2 provides a concise overview of the main challenges limiting the effective development of functional foods, with particular emphasis on the stability of bioactive compounds, sensory quality, consumers’ acceptance, and economic sustainability. These factors collectively determine the feasibility of translating functional food research into commercially viable and scientifically robust products, underscoring the need for integrated technological and formulation strategies.

## 5. Progresses in Functional Food Development

The challenge of directly using bioactive compounds has encouraged efforts to develop advanced delivery systems capable of maintaining the functional integrity of food matrices.

### 5.1. Encapsulation for Controlled Release and Post-Digestion Performance

Microencapsulation has evolved from a technology to a strategy, which can shield labile bioactive compounds against inactivation during food processing and in the GIT, ensuring their controlled delivery and improved bioavailability [67]. The significant sensitivity of bioactive compounds to processing and storage conditions, such as heat treatments, oxygen exposure, light, and pH fluctuations, is one of the main constraints in functional food formulation. Direct fortification frequently results in significant losses in bioactive integrity, which lowers post-digestion efficacy and risks health claims, as numerous studies have shown [64,68].

Microencapsulation has also shown efficiency in protecting bioactive compounds from external stress factors through physical separation. For instance, Szpicer et al. [68] found that microencapsulated phenolic compounds showed greater antioxidant activity than free phenolics after heat treatment. This suggests that microencapsulation protects phenolic compounds from oxidative degradation through the presence of microencapsulation matrices. On the other hand, proteins and polysaccharides act as diffusion barriers that prevent water and oxygen diffusion, hence improving storage stability. Among all, heat-sensitive and water-soluble vitamins are the most susceptible bioactives during processing. Techniques such as spray-drying and freeze-drying have been reported to significantly improve vitamin C retention in fruit powders, while encapsulated forms demonstrate a much lower degradation rate during storage compared to their free forms, as reviewed by Baruroh et al. [69]. All these findings indicate that encapsulation plays a very important role in maintaining the functionality of micronutrients in thermally processed foods.

Encapsulation also allows for considerable value additions to both probiotics and bioactive peptides. Indeed, higher survivability upon consumption is a consequence of increased tolerance of encapsulated probiotic strains to oxygen, heat, and water loss [50,69]. On its part, the enzyme inhibitory and antioxidant activities of the bioactive peptides are well retained since the encapsulation reduces the structural deterioration and minimizes interactions with reactive food components [50].

Despite the well-documented advantages of microencapsulation in enhancing the stability and functionality of bioactive compounds, its performance is highly dependent on the intrinsic properties of the encapsulating materials and the processing parameters employed [67]. In particular, protein-based carriers exhibit strong affinity toward phenolic compounds through hydrogen bonding and hydrophobic interactions, which contribute to improved retention and protection within complex food matrices [67]. In contrast, polysaccharide-based systems, such as alginate, are more suitable for probiotic delivery due to their ability to form structured matrices that limit exposure to oxygen, moisture and environmental stressors [69]. This comparison highlights that encapsulation efficiency is governed not only by protection mechanisms but also by the compatibility between the bioactive compound and the carrier system, emphasizing the need for a tailored rather than universal design approach.

From a technological standpoint, the effectiveness of encapsulation is also influenced by processing conditions, particularly during industrial operations such as spray-drying, which may expose bioactives to thermal stress and compromise structural integrity [67]. Such limitations are especially relevant for heat-sensitive compounds, where even partial degradation can significantly reduce bioactivity and functional performance after digestion [69]. Accordingly, the balance between processing scalability and preservation of functional properties remains a critical challenge in the development of encapsulation systems for real food applications [70].

Overall, these observations indicate that the rational design of encapsulation systems must integrate both material selection and processing optimization to achieve stable, functional, and application-relevant delivery systems for diverse classes of bioactive compounds [67,69].

Table 3 provides a summary of various encapsulation techniques to protect these compounds.

The studies summarized in Table 3 collectively demonstrate that microencapsulation plays a crucial role in improving the stability of diverse classes of bioactive compounds during food processing and storage. Nevertheless, the degree of protection varies considerably depending on both the nature of the encapsulated compound and the physicochemical properties of the carrier matrix. However, despite the reported improvements in stability and retention, most studies remain focused on short-term processing conditions or controlled experimental environments. Consequently, the long-term stability of encapsulated systems in complex food matrices and under industrial storage conditions remains insufficiently explored. These limitations highlight the need for more systematic comparative studies that integrate encapsulation efficiency, storage stability, and functional performance in real food systems in order to better evaluate the practical applicability of encapsulation technologies in functional food development.

### 5.2. Modulation of Gastrointestinal Stability and Bioaccessibility

One of the major hindrances in the translation of the health benefits of bioactive compounds into physiological effects is their instability during gastrointestinal digestion and, hence, degradation during this process. Microencapsulation has been identified as an efficient approach for shielding such sensitive compounds from degradation by gastric juices and bile salts in order to achieve a higher delivery of bioactives in the small intestine in a bioaccessible form. The recovery rate of microencapsulated bioactives has been reported to be higher compared to their free form in in vitro digestion models [67,70].

For instance, the microencapsulation of phenolic extracts from grape pomace has revealed that microencapsulates prepared with sodium alginate and gelatin gel significantly improved the Bioaccessibility Index (BI) values when compared to those without microencapsulation [70]. Ștefănescu et al. reported the protection mechanism using the leaf extract of Vaccinium edible berries, wherein microencapsulation prevented enzymatic breakdown, leading to the highest possible bioactive compound delivery to the intestinal phase [71]. The numerical evidence supporting these findings is presented in Table 4.

The data presented in Table 4 illustrate the impact of microencapsulation on the bioaccessible fraction of phenolic bioactives, making them more amenable to cell interaction and binding. These findings demonstrate the efficacy of using a combination of biopolymers, such as polysaccharides and proteins, as an encapsulation matrix compared to the use of a single material. The importance of choosing appropriate wall materials to facilitate the controlled release of bioactives within the digestive tract is evident [10]. However, despite these promising results regarding enhanced bioaccessibility, it is crucial to critically evaluate the translational potential of these methodologies. Most cited studies utilize static in vitro digestion models, such as the INFOGEST protocol, which may not fully replicate the dynamic hormonal and enzymatic fluctuations of the human gastrointestinal tract [9]. Furthermore, technological scalability remains a significant bottleneck; for instance, while spray-drying is widely adopted due to its cost-efficiency, the thermal stress during processing can diminish the potency of sensitive bioactives [69]. In contrast, emerging hybrid biopolymer matrices offer superior protection but often involve complex fabrication processes that increase production costs [67]. Future research must prioritize in vivo validation and sensory analysis to ensure that improved bioactivity does not come at the expense of consumer acceptance or industrial economic viability [42].

While conventional microencapsulation techniques such as spray-drying, freeze-drying, and alginate-based systems have demonstrated considerable effectiveness in protecting bioactive compounds during processing and digestion, they primarily function as passive protective barriers that limit degradation caused by environmental stresses [67]. In response to these limitations, recent research has increasingly focused on more advanced delivery strategies that extend beyond simple physical protection. Emerging approaches include nano-encapsulation platforms, pH-responsive carriers, and hybrid biopolymer matrices designed to achieve more precise control over release behavior and gastrointestinal targeting. Unlike traditional encapsulation systems, these advanced carriers can respond to environmental triggers such as pH variations or enzymatic activity, thereby improving the stability and site-specific delivery of sensitive bioactives within the gastrointestinal tract [64].

Such developments are particularly relevant for compounds with inherently low bioavailability, including polyphenols and certain bioactive peptides, where controlled intestinal release may enhance physiological efficacy. However, despite these technological advances, important questions remain regarding their industrial scalability, economic feasibility, and long-term safety, highlighting the need for balanced evaluation that considers both technological innovation and practical applicability in functional food systems [68].

Encapsulation has been widely reported to enhance the gastrointestinal stability and bioaccessibility of bioactive compounds; however, its effectiveness is strongly influenced by the physicochemical characteristics of both the encapsulated compound and the carrier matrix [70]. From a comparative perspective, alginate-based systems are particularly effective due to their pH-responsive behavior, which enables protection under the acidic gastric environment and facilitates controlled release under intestinal conditions. In contrast, protein-based carriers may offer stronger molecular interactions with certain bioactives, yet their susceptibility to enzymatic degradation can limit their protective capacity during gastrointestinal transit [71]. This contrast underscores that the functional performance of encapsulation systems is not universal but rather matrix-dependent, requiring careful alignment between the properties of the bioactive compound and the encapsulation material.

Although in vitro digestion models frequently report improved bioaccessibility, including higher Bioaccessibility Index (BI) values, such findings do not always accurately reflect in vivo conditions, where complex physiological factors such as peristalsis, enzymatic variability, and food matrix interactions play a significant role [9,70]. This limitation highlights a critical gap between controlled experimental systems and real physiological environments, raising questions about the translational relevance of in vitro results. Moreover, inconsistencies in reported release kinetics and bioaccessibility across different studies further emphasize the variability inherent to encapsulation systems, particularly when different materials, methods, and experimental conditions are employed.

Overall, while encapsulation provides a promising strategy for improving intestinal delivery, its true efficacy must be evaluated through more physiologically relevant models and standardized methodologies to ensure reliable prediction of its behavior in real food systems and biological contexts [70].

### 5.3. Controlled Release and Targeted Delivery

Microencapsulation of functional foods can be one of the most advantageous technologies concerning the control of bioactive compound delivery in different parts of the GIT [72]. This approach not only protects sensitive compounds from a harsh environment but also delivers these compounds to specific physiological locations, such as the intestine or the colon, where absorption can efficiently occur [73].

In particular, alginate–pectin matrices have shown great interest owing to their pH responsiveness and potential use in controlled release delivery of phenolic compounds. The biopolymeric complexes are more stable in an acidic gastric environment, which swells in response to the close-to-neutral pH in the small intestine. The use of alginate–pectin microcapsules prepared by extrusion to encapsulate papaya polyphenols improved the protection of polyphenols during the gastric phase. This facilitated their release during the intestinal phase, increasing their bioaccessibility as opposed to non-encapsulated polyphenols [74]. This shows the advantage of using polysaccharide-based composites in enhancing the delivery and utilization properties of phenolic compounds [75].

Controlled release also plays an important role in probiotics. The layer-by-layer encapsulation method effectively protects the viability of probiotics during gastric transit. Chehreara et al. reported that multilayer-coated microcapsules can optimize the delivery of *Lacticaseibacillus rhamnosus*, maximizing its functional activity [76]. Similarly, Saiz-Gonzalo et al. found microencapsulation of pea protein resulted in enhanced survivability of probiotic bacteria in gastric simulations, with improved delivery profiles when compared to unencapsulated cells [77]. Moreover, modern systems also have enzyme- or bile-sensitive parts, such as alginate–pectin hydrogels, targeted specifically at the intestinal milieu [78,79]. Thus, by closing the gap between physicochemical stability and activity, microencapsulation ensures that biologically active compounds can reach intestinal epithelial cells as an intact absorbable entity [9]. The studies summarized in Table 5 illustrate the efficacy of various encapsulation techniques to improve the targeted release of bioactives.

These reports prove microencapsulation is an efficient technique for the controlled release of bioactive components in functional foods. Hence, microencapsulation assumes important functions in ensuring the maximal biological efficacy of functional foods by delivering bioactive components in the desired form to the target site in the human body.

The ability of encapsulation systems to achieve controlled and targeted release is largely determined by the composition and structural characteristics of the delivery matrix [74]. From a comparative standpoint, polysaccharide-based systems, such as alginate–pectin composites, exhibit superior performance in pH-responsive release, particularly for the targeted delivery of phenolic compounds to the intestinal phase, owing to their ability to form stable gel networks that resist acidic gastric conditions. In contrast, protein-based systems may provide enhanced molecular interactions with bioactive peptides; however, their structural susceptibility to enzymatic degradation can result in less predictable and less controlled release behavior [75]. This distinction highlights that release performance is not solely governed by encapsulation efficiency but is strongly influenced by the interaction between the carrier material and the encapsulated compound.

In comparison, advanced delivery systems such as layer-by-layer coatings offer improved control over probiotic delivery by enhancing resistance to gastric conditions and enabling more sequential and regulated release profiles [76]. Nevertheless, despite these functional advantages, such systems are often associated with increased technological complexity and higher production costs, which limit their scalability and practical implementation in large-scale food production. Furthermore, inconsistencies in release kinetics across different encapsulation strategies further complicate their application, particularly when translating laboratory-optimized systems into industrial contexts.

Overall, these observations indicate that although controlled release technologies significantly improve the functional performance of bioactive compounds, their practical adoption requires a careful balance between release precision, processing complexity, and economic feasibility [74,76].

### 5.4. Enhancement of Post-Digestion Biological Activity

Microencapsulation protects bioactive compounds from degradation in the GI tract, securing their bioactive potential once they are released in the GIT. Evidence from both in vitro and animal models has shown that microencapsulated bioactives retain their functions and, in addition, sometimes have higher functions than their free counterparts [72]. For example, encapsulation of phenolic extracts of Ciriguela (*Spondias purpurea*) peel performed using spray-drying or freeze-drying methods increased the stability of total phenolic content significantly after the digestion, compared to the free form of extracts [80]. The encapsulation of phenolic compounds from chia sprouts showed an increase in bioavailability under simulated intestinal environments, stronger antioxidant activity, and additional antibacterial and antidiabetic actions compared with the non-encapsulated form of phenolics [81]. Encapsulated peptides subjected to simulated gastrointestinal conditions maintained greater inhibitory activity against key metabolic enzymes, including angiotensin-converting enzyme (ACE) and α-glucosidase, compared to their non-encapsulated forms [18]. Studies have proved (Table 6) that encapsulation has the ability to preserve and improve the functionality of bioactive compounds even after digestion.

Beyond protection against environmental degradation, microencapsulation enhances the bioavailability of bioactive compounds through several physicochemical mechanisms. Encapsulation matrices composed of polysaccharides, proteins, or lipid-based carriers can establish hydrogen bonding, electrostatic interactions, and hydrophobic associations with phenolic molecules, thereby stabilizing their chemical structure during gastrointestinal digestion [72,79]. These interactions help reduce premature oxidation and enzymatic degradation during the gastric phase while enabling a more controlled release of the bioactive compounds in the intestinal environment, where absorption primarily occurs [36,39]. Furthermore, encapsulation systems may improve the apparent solubility and dispersion of poorly soluble bioactives, facilitating their interaction with intestinal epithelial cells and increasing their bioaccessibility [36]. Such mechanisms are particularly relevant for phenolic compounds, which often exhibit low intrinsic bioavailability due to rapid degradation and limited intestinal permeability. Consequently, microencapsulation strategies have been widely explored as delivery systems to enhance the stability, controlled release, and functional efficacy of dietary bioactives in functional foods and nutraceutical formulations [72,79].

**Figure 1:**
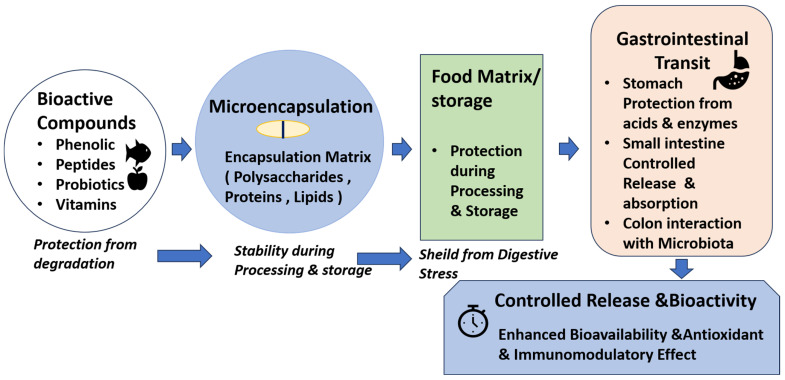
Microencapsulation and protection of bioactive compounds in functional foods.

Figure 1 illustrates the fundamental process of microencapsulation of bioactive compounds in functional foods, highlighting protection during food processing and storage, gastrointestinal transit, and targeted release to enhance bioavailability and post-digestion biological activity.

As illustrated in the figure, microencapsulation provides a multi-layered protective system for bioactive compounds such as phenolics, vitamins, peptides, and probiotics against environmental stresses during processing and storage. During gastrointestinal transit, the encapsulating matrix minimizes degradation by gastric acids and digestive enzymes, enabling controlled release in the small intestine and colon, where absorption or interaction with the gut microbiota occurs. This approach not only ensures chemical stability but also enhances bioaccessibility, antioxidant activity, and immunomodulatory potential, highlighting the critical role of microencapsulation in designing effective and functional food products.

Encapsulation has been reported to enhance the post-digestion biological activity of bioactive compounds by preserving their structural integrity and improving their functional availability following gastrointestinal digestion [80]. In comparison to their free counterparts, encapsulated bioactives generally demonstrate higher retention of functional properties, including antioxidant, antihypertensive, and antimicrobial activities, particularly when protected within polysaccharide- or protein-based matrices that limit degradation during digestion [80,81]. This comparative advantage, however, is highly dependent on both the encapsulation technique and the intrinsic properties of the bioactive compound, indicating that the observed functional enhancement is not universally guaranteed across all systems.

From a technological perspective, different encapsulation methods exhibit distinct advantages and limitations. For instance, spray-drying is widely applied due to its scalability and cost-effectiveness, yet it may induce partial degradation of heat-sensitive compounds due to thermal exposure, potentially compromising functional integrity. In contrast, freeze-drying offers superior preservation of sensitive bioactives but is associated with higher energy consumption and production costs, limiting its industrial applicability [80,82]. This trade-off highlights the ongoing challenge of balancing functional preservation with technological feasibility.

Importantly, despite the frequent observation of improved intestinal bioaccessibility, several studies report limited or inconsistent improvements in systemic bioavailability, suggesting that enhanced digestion does not always translate into proportional physiological outcomes [83]. This discrepancy points to the complexity of post-digestive processes, including absorption, metabolism, and transport, which are not fully captured by conventional in vitro models.

Overall, these findings emphasize the need for a more critical and mechanistic evaluation of encapsulation systems, with particular attention to their metabolic fate and actual physiological relevance, rather than relying solely on in vitro indicators of enhanced functionality [80,83].

### 5.5. Sensory and Quality Advantages

It has been found that large amounts of certain bioactive compounds, such as hydrophobic phenolics and peptides, are frequently linked with unwanted sensory properties such as bitterness, astringency, and off-flavors. Microencapsulation has a vital role to play in influencing the sensory acceptability of FFUs; Duque-Soto et al. evaluated the gastrointestinal stability of phenolic compounds of olive leaf by co-administration and microencapsulation with non-digestible carbohydrates [82]. The inclusion of probiotic-microencapsulated yogurt and cheeses has ensured that the bacterial viability was unaffected and did not impair the sensory properties. Therefore, microencapsulation serves as a multidimensional technology combining nutrition and sensory properties [84]. Microencapsulation can also improve sensory quality and acceptability by consumers of functional foods (Table 7) by reducing bitterness and masking astringency, while the stabilization of flavor compounds enables the integration of bioactive ingredients into products. Assuring no compromise in taste, texture, or overall sensory appeal supports the successful development of functional products.

Large amounts of certain bioactive compounds, especially hydrophobic phenolics and peptides, are often associated with undesirable sensory properties such as bitterness and astringency. Sensory challenges are typically assessed using trained sensory panels, hedonic scoring, and descriptive analysis techniques to quantify taste, mouthfeel, and overall acceptability [85,86]. Microencapsulation has been shown to mitigate these effects by masking unpleasant tastes, controlling release rates, and stabilizing flavor compounds [68,82]. For example, oxidized starch hydrogel microencapsulation of proanthocyanidins reduced perceived astringency in beverages [85], while spray-drying polyphenols from cocoa shells with maltodextrin lowered bitterness and improved sensory scores [86]. Co-microencapsulation with inulin for olive leaf phenolics stabilized color and reduced flavor alterations, maintaining overall consumer acceptability [82]. These studies demonstrate that microencapsulation not only preserves bioactivity but also addresses sensory issues through controlled release and physical separation of compounds from taste receptors.

The studies summarized in Table 7 highlight how microencapsulation techniques can enhance both sensory attributes and overall quality of bioactive compounds in functional foods. For instance, the use of oxidized starch hydrogel in beverages reduced bitterness and astringency, thereby improving consumer acceptability [85], while spray-drying with maltodextrin enhanced the sensory performance of cocoa polyphenols in chocolate products [86]. These findings suggest that the choice of encapsulation system should be tailored to the type of bioactive compound and the food matrix. General polyphenols and alkaloids can benefit from broad microencapsulation strategies that improve flavor delivery and mask undesirable tastes [68], whereas co-encapsulation with inulin for olive leaf phenolics stabilized color and minimized flavor alterations [82]. For probiotics, alginate–protein microcapsules maintained texture and taste while increasing consumer acceptance [84]. Overall, these results indicate that microencapsulation not only protects bioactive compounds from chemical degradation but also plays a critical role in optimizing sensory experiences, which is essential for the successful market adoption of functional foods.

Encapsulation technologies have emerged as a versatile strategy to enhance the stability, bioaccessibility, and functional performance of bioactive compounds in food systems; however, their effectiveness varies significantly depending on the physicochemical nature of the encapsulated compound and the structural characteristics of the carrier system [72]. A comparative analysis of different bioactive classes highlights that phenolic compounds primarily benefit from protection against oxidation and controlled release mediated by hydrogen bonding and hydrophobic interactions with polymeric matrices, whereas peptides require encapsulation systems that effectively limit enzymatic degradation during gastrointestinal transit [72,76]. In contrast, probiotics depend on structural systems that ensure survival under acidic gastric conditions and enable viable release in the intestinal environment, while vitamins benefit from lipid- or polysaccharide-based systems that improve their stability against heat, light, and oxidation [69,82]. These distinctions emphasize that encapsulation strategies must be tailored to the specific functional and structural requirements of each bioactive compound rather than relying on a universal approach.

Despite the demonstrated improvements in stability and bioaccessibility under in vitro conditions, a persistent limitation remains in the translation of these findings to in vivo systems, where complex physiological factors such as digestion kinetics, food matrix interactions, and individual variability significantly influence the fate of encapsulated compounds [70]. This discrepancy highlights the need for caution when extrapolating laboratory results to biological systems and underscores the importance of integrating physiologically relevant models in encapsulation research [70]. Additionally, variability in release behavior among different encapsulation systems further complicates their practical application, as systems designed for controlled release may exhibit incomplete or inconsistent bioactive delivery under certain conditions [78].

From an application standpoint, a critical challenge lies in balancing encapsulation efficiency with scalability and cost-effectiveness. While advanced systems such as multilayer coatings and responsive biopolymer matrices provide enhanced control over release and protection, their industrial application is often limited by processing complexity and economic constraints [80]. Conversely, widely used techniques such as spray-drying offer better scalability but may compromise the stability of heat-sensitive bioactives if not carefully optimized, illustrating the trade-off between technological performance and industrial feasibility [82].

Moreover, the current lack of standardized methodologies for evaluating encapsulation performance—both in vitro and in vivo—represents a significant barrier to comparing results across studies and advancing the field toward practical applications [9,70]. Addressing this limitation requires the development of harmonized protocols that can reliably assess the stability, release behavior, and bioavailability of encapsulated compounds under physiologically relevant conditions.

Overall, while encapsulation provides a powerful platform for improving the delivery and functionality of bioactive compounds, its successful application depends on a rational design approach that considers the interplay between bioactive properties, carrier systems, processing conditions, and physiological constraints [72,79]. Future research should therefore focus on optimizing encapsulation systems with respect to both mechanistic performance and real-world applicability, thereby facilitating the translation of these technologies into effective and reliable functional food products [82].

## 6. Challenges and Future Directions

Microencapsulation offers clear advantages for enhancing the stability, controlled release, and bioavailability of bioactive compounds in functional foods [10,66]. However, translating these laboratory-scale innovations into industrial applications presents a series of interrelated challenges. Technically, high costs associated with encapsulation materials and processing methods, combined with variable release profiles under gastrointestinal conditions, limit the feasibility of large-scale production [64,79]. Moreover, differences in encapsulation efficiency across bioactive compounds necessitate rigorous optimization and quality control protocols to achieve reproducible outcomes [67,68].

Current research efforts aim to address these limitations by exploring sustainable biopolymers, green solvents, and eco-friendly matrices, reflecting the growing consumer demand for “clean-label” functional foods [10,66]. Additionally, synergistic strategies combining specific encapsulation techniques with targeted bioactive compounds are being investigated to simultaneously improve stability, functionality, and post-digestion bioavailability [64,67]. Such integrated design approaches are essential to bridge the gap between promising experimental results and scalable industrial processes [66,68].

Despite in vitro evidence demonstrating improved protection and bioaccessibility of polyphenols, bioactive peptides, and probiotics, in vivo validation remains limited [10,66,72]. For example, Motilva et al. examined the bioavailability of red wine polyphenols enriched with either free or nano-encapsulated extracts. The study found that while certain metabolites (e.g., syringic acid salts, malvidin-3-O-glucoside) were excreted at higher levels, overall absorption was not universally enhanced, highlighting the complex interplay of human metabolism with encapsulation strategies [87]. Similarly, Mueller et al. (2018) reported that whey-protein-based microcapsules increased urinary excretion of anthocyanins but did not improve plasma concentrations, whereas pectin-based matrices enhanced intestinal accessibility by 24% without significantly affecting systemic bioavailability [83]. These findings emphasize the need for further clinical studies to optimize dosage forms, evaluate release kinetics under physiological conditions, and establish the functional efficacy of encapsulated bioactives in humans [83,87].

Economic feasibility also remains a critical barrier. While laboratory-scale studies often utilize premium biopolymers and energy-intensive methods, these approaches may be unsustainable at an industrial scale. Potential cost-reduction strategies, including abundant, low-cost biopolymers (e.g., starch, alginate, cellulose derivatives) and energy-efficient or solvent-free encapsulation methods, must be critically assessed for their impact on encapsulation efficiency, controlled release, and bioactive functionality [64,79]. Without rigorous evaluation of these trade-offs, the health-promoting benefits of functional foods could be compromised [10,66].

Finally, regulatory compliance represents an additional layer of complexity. Functional foods incorporating encapsulated bioactives must adhere to guidelines set by regional authorities such as the European Food Safety Authority (EFSA) and the U.S. Food and Drug Administration (FDA), which cover safety assessment, labeling, and permissible health claims [1,63]. Early integration of regulatory considerations is crucial, as compliance influences formulation decisions, choice of encapsulation materials, and substantiation of functional claims [2,63]. Failure to align product development with regulatory frameworks may limit their commercialization potential despite technological success [63].

In addition to technical and biological challenges, the environmental impact of large-scale microencapsulation processes represents a critical yet often underemphasized concern. Conventional methods, particularly energy-intensive techniques such as spray-drying or freeze-drying, can generate significant carbon footprints and solvent waste, raising sustainability questions for industrial translation [64,79]. While the adoption of sustainable biopolymers, green solvents, and eco-friendly matrices has been proposed, systematic evaluation of their lifecycle impact, scalability, and effect on encapsulation efficiency remains limited [10,66]. Therefore, advancing microencapsulation for functional foods requires an integrated approach that balances bioactive stability, cost-effectiveness, and environmental responsibility, emphasizing the need for innovation in low-energy, solvent-free, and recyclable encapsulation technologies.

In summary, while micro and nano-encapsulation hold considerable promise for improving the functional and nutritional quality of foods, their practical application requires a multidimensional approach. Technical optimization, clinical validation, cost-effectiveness, sustainability, and regulatory alignment must be addressed collectively to ensure that these innovations translate into real-world health benefits.

## 7. Encapsulation Safety: Critical Perspective

Safety is a fundamental consideration in selecting appropriate encapsulation strategies. Micro and macroencapsulation commonly use biocompatible polymers such as gelatin, alginate, starch, and casein, which are generally recognized as safe (GRAS) for food applications [67,68]. These carriers protect sensitive bioactives—such as polyphenols, fatty acids, and probiotics—against degradation during processing and gastrointestinal transit [70,71]. Macroencapsulation, with its simpler structure, is especially effective for fat-soluble vitamins and carotenoids, providing protection from oxidation and light-induced degradation while maintaining regulatory compliance [69,74].

Nano-encapsulation offers superior bioavailability and targeted delivery but introduces unique safety concerns due to particle size-dependent interactions. Nanoparticles can cross cellular barriers, potentially inducing oxidative stress, inflammation, or unintended uptake of contaminants—the so-called “Trojan horse” effect [88]. While biodegradable food-grade nanocarriers are metabolized similarly to conventional nutrients, long-term effects, including microbiome alterations or tissue accumulation, remain underexplored [88].

Regulatory frameworks emphasize the evaluation of particle size, digestibility, and chronic exposure for novel food ingredients [67,68]. Therefore, while micro and macroencapsulation generally provide safe profiles, nano-formulations require careful design and chronic exposure assessments relative to the bioactive compound [41,42]. Integrating performance optimization with safety evaluation is essential to develop functional foods that deliver bioactive efficacy without compromising consumer health [42,43].

While micro and macroencapsulation generally ensure protection of bioactive compounds, it is important to recognize that the encapsulation matrices themselves may influence the functional efficacy of the encapsulated molecules. Certain polymers or processing conditions can partially degrade sensitive compounds or modify their release kinetics, leading to reduced bioactivity upon ingestion [67,70]. Nano-encapsulation, although providing enhanced targeting and potential bioavailability, introduces additional uncertainties, as interactions between nanoparticle surfaces and bioactives may alter molecular stability or trigger premature release under gastrointestinal conditions [41,42]. Moreover, the long-term consequences of chronic consumption of encapsulated formulations, particularly at the nanoscale, remain insufficiently characterized, raising questions about potential impacts on metabolism, gut microbiota, or systemic distribution [42,43]. These considerations underscore the need for a holistic evaluation that integrates encapsulation performance with comprehensive safety and efficacy assessments, ensuring that functional foods achieve their intended health benefits without compromising bioactive integrity or consumer safety.

## 8. Conclusions

Bioactive compounds undeniably play a central role in the design of functional foods; however, their potential is frequently compromised by instability, low bioaccessibility, and unpredictable behavior within complex food matrices and the gastrointestinal tract. While incorporating vitamins, probiotics, polyphenols, and peptides offers considerable health promise, current applications often overstate efficacy based on in vitro observations that may not translate to meaningful human outcomes. The existing literature highlights methodological limitations, inconsistent reporting of bioavailability, and insufficient integration of sensory and technological constraints, which together hinder practical implementation.

Importantly, the effectiveness of these compounds is highly dependent on their chemical nature, where polyphenols, vitamins, peptides, and probiotics exhibit distinct behaviors in terms of stability, release kinetics, and post-digestion functionality. In this context, microencapsulation provides a tailored approach to enhance the stability of heat- and oxygen-sensitive vitamins, improve the controlled release and intestinal bioaccessibility of polyphenols, and increase the viability and functional activity of probiotics and bioactive peptides.

Despite advances such as microencapsulation and nano-formulations, the field remains largely experimental. Many technologies show promise in controlled settings but fail to address industrial-scale feasibility, cost-effectiveness, and regulatory compliance. Moreover, the interactions between multiple bioactives, their synergistic or antagonistic effects, and their long-term physiological impacts remain poorly understood. There is also a critical lack of standardized protocols to evaluate the stability, release kinetics, and functional efficacy of these compounds across diverse food systems.

Furthermore, the performance of encapsulated systems is strongly influenced by the composition of the food matrix, as interactions with proteins, lipids, and polysaccharides can significantly affect both protection and release behavior. For instance, protein-based matrices may enhance the stabilization of phenolic compounds through molecular interactions, whereas polysaccharide-based systems are more effective in protecting probiotics against harsh gastrointestinal conditions.

Future research must move beyond descriptive studies to mechanistic and translational approaches. Key research priorities include the development of standardized evaluation protocols, integration of in vitro and in vivo models, and systematic assessment of long-term health outcomes. Emphasis should be placed on understanding the fate of bioactives post-ingestion and optimizing delivery systems for controlled release. Emerging technological directions, such as smart delivery systems, nano-enabled carriers, and stimuli-responsive encapsulation matrices, offer promising opportunities to improve targeted release and functional efficacy.

However, despite these promising advances, several technological and practical barriers remain, including limited scalability, high production costs, variability in release profiles under physiological conditions, and the lack of standardized assessment methods. In addition, potential impacts on sensory properties and regulatory challenges must be carefully considered to ensure successful industrial application. Bridging the gap between laboratory-scale innovations and real-world functional foods requires interdisciplinary efforts that integrate food chemistry, digestive physiology, sensory science, and industrial engineering. By addressing these challenges, the field can progress from conceptual promise toward functional foods that consistently deliver measurable health benefits.

## Figures and Tables

**Table 1:** Major bioactive compounds in functional foods, their post-digestion fate, and biological activities.

Bioactive Compound	Main Food Sources	Post-Digestion Fate	Key Biological Activities
Phenolic Compounds	Fruits, vegetables, Whole grains Tea, olive oil [5,6]	Partial release during digestion [7] Metabolites formed by gut microbiota [15]	Antioxidant signaling [5] Anti-inflammatory effects (IL-6, TNF-α) [12] Modulation of glucose and lipid metabolism [15,16]
Bioactive Peptides	Dairy products Soy Cereals, legumes [16]	Released during digestion, or fermentation [16] Activity depends on sequence and enzyme resistance [17]	ACE inhibition Blood pressure regulation [16] Antioxidant activity [17] Immunomodulation Glucose metabolism [19]
Probiotics	Yogurt Milk-based kefir cheese Fermented vegetables [24,25,26,27]	Partial survival through gastric and intestinal phases Metabolites and cell components remain bioactive [24,26]	Immune modulation [26] Gut barrier reinforcement [27] Pathogen inhibition [25] SCFAs production Metabolic regulation [28]
Vitamins (Water- and Fat-Soluble)	Fruits, vegetable Dairy Fortified foods [36]	Antioxidant defense (C, E) Immune regulation (A, D) Energy metabolism (B-complex) [37,38]	Antioxidant defense (C, E) [36] Immune regulation (A, D) [37] Energy metabolism (B-complex) [33]

Abbreviations: ACE—Angiotensin-Converting Enzyme, IL-6—Interleukin-6, TNF-α—Tumor Necrosis Factor-alpha, SCFAs—Short-Chain Fatty Acids.

**Table 2:** The key challenges in functional food development.

Challenge Category	Description	Impact on Functional Food
Consumer perception	Skepticism toward health claims and limited awareness of scientific evidence	Reduced consumer acceptance and market penetration [62]
Economic and sustainability issues	High production costs and sustainability concerns in sourcing bioactive compounds	Limited affordability and scalability [63]
Processing stability	Degradation of vitamins and phenolic compounds due to heat, oxygen, and pH	Reduced bioactivity and functional efficacy [36,64]
Sensory quality	Development of bitterness, color changes, or off-flavors	Negative impact on sensory acceptance [65]
Safety and efficacy validation	Need for scientific validation and regulatory compliance	Challenges in commercialization and health claims [66]

**Table 3:** Protection of bioactive compounds during processing and storage.

Bioactive Compound	Processing/Storage Challenge	Encapsulation Strategy	Observed Outcomes	Quantitative Indicators
Phenolic compounds	Thermal processing, oxygen exposure	Protein/polysaccharide microcapsules	Improved antioxidant stability	Phenolic retention > 80% after thermal treatment [68]
Vitamins (Vitamin C)	Oxidation during processing and storage	Spray-drying/freeze-drying	Reduced degradation during storage	Encapsulation efficiency > 90% [69]
Probiotics	Oxygen exposure, dehydration, temperature fluctuations	Alginate/protein microcapsules	Improved survival during storage and digestion	Encapsulation yield 73–94%; viability maintained during storage [69]
Bioactive peptides	Structural degradation and interactions with food matrix	Protective microencapsulation coatings	Preserved enzyme-inhibitory and antioxidant activity	Improved activity retention during storage [18]
Vitamin C	Thermal and oxidative degradation	Alginate–gum arabic spray-dried microcapsules	Enhanced thermo-oxidative stability	Encapsulation efficiency > 90% [69]
Probiotic LAB	Storage and gastrointestinal stress	Alginate–chitosan double coating	Higher viability during long-term storage	Survival maintained for up to 6 months; EY up to 94% [18]

**Table 4:** Effect of microencapsulation on bioaccessibility of phenolic compounds after simulated gastrointestinal digestion.

Bioactive Source	Encapsulation Technique/Coating	Bioaccessibility Index (BI)—Encapsulated	Bioaccessibility Index (BI)—Non-Encapsulated	Key Findings and Interpretation
Grape pomace extract	Alginate + gelatin spray-dried microcapsules	37.8–96.2% (total phenolics); individual phenolic BI up to 2028.7%	Lower BI across phenolics	Encapsulation protected phenolic compounds from degradation during gastric digestion, resulting in enhanced intestinal release and significantly improved bioaccessibility [70]
Grape pomace extract	Alginate + gum Arabic or SA microcapsules	25.2–82.4% (total phenolics)	18.9–23.9% (control)	Biopolymer combinations improved phenolic stability and release behavior compared with non-encapsulated extracts, highlighting the importance of matrix composition in delivery systems [70]
*Vaccinium vitis-idaea* leaf extract	Maltodextrin-based microcapsules	45.43% BI	38.65% (non-encapsulated VCS sample)	Microencapsulation improved the stability of phenolic compounds during simulated digestion, leading to increased bioaccessible fractions [71]
*Vaccinium corymbosum* leaf extract	Maltodextrin-based microcapsules	41.07% BI	38.65%	Encapsulation enhanced phenolic availability after digestion, suggesting improved delivery efficiency within gastrointestinal conditions [71]
*Ciriguela peel* extract	Spray-dried microcapsules	42.30%	28.70%	Microencapsulation significantly improved phenolic stability during digestion and enhanced bioaccessibility, confirming the protective effect of the encapsulation matrix [64,70].
*Olive leaf phenolic* extract	Alginate + non-digestible carbohydrate microcapsules	48.50%	33.20%	Co-encapsulation with carbohydrates enhanced gastrointestinal stability and bioaccessibility of phenolics compared with non-encapsulated extract [64,71].

**Table 5:** Controlled release of bioactive compounds via microencapsulation.

Bioactive	Encapsulation System	Target Release Trigger	Outcome
Quercetin	Alginate–chitosan–inulin microspheres	pH shift (intestine)	Enhanced release at intestinal pH vs. gastric [75]
*L rhamnosus*	Layer-by-layer chitosan/alginate coating	pH shift	Improved survival through gastric + intestinal release [76]
Probiotic cells	Pea protein microcapsules	pH + enzyme digestion	Higher viability in intestinal phase [77]
Quercetin/Phenolic extract	Alginate–pectin microcapsules	pH shift (intestine)	Controlled intestinal release and improved stability [74]
Bioactive extract	Emulsion-templated plant protein microcapsules	pH + enzymatic digestion	Enhanced digestibility and enteric release [73]

**Table 6:** Post-digestion biological activity of encapsulated bioactive compounds.

Bioactive Compound	Source Food Matrix	Encapsulation System	Simulated Digestion Model	Post-Digestion Biological Effect	Potential Functional Food Application
Phenolic extract	Ciriguela peel	Spray-dried/Freeze-dried	In vitro gastrointestinal digestion	Higher retention of total phenolics and enhanced antioxidant activity compared with free extract [80]	Potential incorporation into antioxidant-enriched beverages or nutraceutical formulations [11,79]
Phenolic extract	Chia sprouts	Polysaccharide-based microcapsules	Simulated intestinal digestion	Improved bioavailability and enhanced antioxidant, antibacterial, and antidiabetic activities [81]	Development of functional foods targeting metabolic health [11,64]
Bioactive peptides	Whey/Casein	Spray-dried with maltodextrin or protein carriers	Simulated gastrointestinal digestion	Maintained ACE-inhibitory activity and enzyme stability after digestion [18]	Application in functional dairy products or cardiometabolic health supplements [11,79]
Phenolic extract	Plant protein-based emulsion	Emulsion-templated plant protein microcapsules	Simulated gastrointestinal digestion	Enhanced digestibility and controlled release in intestinal phase [73]	Potential inclusion in functional foods and nutraceuticals [11,64]

**Table 7:** Sensory and quality advantages of microencapsulated bioactives in functional foods.

Bioactive/Target	Food Matrix	Encapsulation System/Strategy	Sensory/Quality Benefit
Phenolic compounds (e.g., proanthocyanidins)	Beverages	Oxidized starch hydrogel microencapsulation	Masked astringency and bitterness; increased perceived acceptability [85]
Polyphenols from cocoa shell	Chocolate bars	Spray-dried with maltodextrin	Reduced bitterness and astringency; good maintained sensory scores [86]
General polyphenols/alkaloids	Various foods	Microencapsulation	Masked undesirable tastes, improved flavor delivery and palatability [68]
Olive leaf phenolics	Functional beverage	Co-microencapsulation with inulin	Stabilized color; reduced flavor alterations [82]
Probiotics	Yogurt/Cheese	Alginate/protein microcapsules	Maintained texture and taste; improved consumer acceptance [84]
Phenolic extracts	Bakery products/Cookies	Spray-dried with maltodextrin or protein carrier	Reduced bitterness, maintained color and texture [48,49]
Plant-based bioactive peptides	Plant-based bioactive peptides	Emulsion-templated plant protein microcapsules	Enhanced mouthfeel, no off-flavor, improved palatability [68,82]
Grape pomace phenolics	Fruit juice	Alginate-based microcapsules	Maintained color and aroma, reduced bitterness [70]

## Data Availability

No new data were created or analyzed in this study.

## References

[1] Temple N.J. (2022). A rational definition for functional foods: A perspective. Front Nutr..

[2] Butnariu M., Sarac I. (2019). Functional food. Int. J. Nutr..

[3] Kobayashi E., Sato Y., Umegaki K., Chiba T. (2018). Analysis of safety alerts associated with dietary supplements from Japan and overseas. Shokuhin Eiseigaku zasshi. J. Food Hyg. Soc. Jpn..

[4] Zheng Y., Qin C., Wen M., Zhang L., Wang W. (2024). The effects of food nutrients and bioactive compounds on the gut microbiota: A comprehensive review. Foods.

[5] Han X., Shen T., Lou H. (2007). Dietary polyphenols and their biological significance. Int. J. Mol. Sci..

[6] El-Saadony M.T., Saad A.M., Mohammed D.D.M., Alkafaas S.S., Abd El Mageed T.A., Fahmy M.A., Ezzat Ahmed A., Algopishi U.B., Abu-Elsaoud A.M., Mosa W.F. (2025). Plant bioactive compounds: Extraction, biological activities, immunological, nutritional aspects, food application, and human health benefits-A comprehensive review. Front. Nutr..

[7] Essa M.M., Bishir M., Bhat A., Chidambaram S.B., Al-Balushi B., Hamdan H., Govindarajan N., Freidland R.P., Qoronfleh M.W. (2023). Functional foods and their impact on health. J. Food Sci. Technol..

[8] Lippolis T., Cofano M., Caponio G.R., De Nunzio V., Notarnicola M. (2023). Bioaccessibility and bioavailability of diet polyphenols and their modulation of gut microbiota. Int. J. Mol. Sci..

[9] Minekus M., Alminger M., Alvito P., Ballance S., Bohn T., Bourlieu C., Carrière F., Boutrou R., Corredig M., Dupont D. (2014). A standardised static in vitro digestion method suitable for food–an international consensus. Food Funct..

[10] Dahiya D., Terpou A., Dasenaki M., Nigam P.S. (2023). Current status and future prospects of bioactive molecules delivered through sustainable encapsulation techniques for food fortification. Sustain. Food Technol..

[11] Arshad Z., Shahid S., Hasnain A., Yaseen E., Rahimi M. (2025). Functional foods enriched with bioactive compounds: Therapeutic potential and technological innovations. Food Sci. Nutr..

[12] Rodríguez-Romero J.d.J., Arce-Reynoso A., Parra-Torres C.G., Zamora-Gasga V.M., Mendivil E.J., Sáyago-Ayerdi S.G. (2023). In vitro gastrointestinal digestion affects the bioaccessibility of bioactive compounds in Hibiscus sabdariffa beverages. Molecules.

[13] Maleki S.J., Crespo J.F., Cabanillas B. (2019). Anti-inflammatory effects of flavonoids. Food Chem..

[14] Ávila-Gálvez M.Á., Giménez-Bastida J.A., Karadeniz B., Romero-Reyes S., Espín J.C., Pelvan E., González-Sarrías A. (2024). Polyphenolic Characterization and Anti-Inflammatory Effect of In Vitro Digested Extracts of *Echinacea purpurea* L. Plant Parts in an Inflammatory Model of Human Colon Cells. Int. J. Mol. Sci..

[15] Sharma H., Anand A., Halagali P., Inamdar A., Pathak R., Taghizadeh-Hesary F., Ashique S. (2024). Advancement of nanoengineered flavonoids for chronic metabolic diseases. Role of Flavonoids in Chronic Metabolic Diseases: From Bench to Clinic.

[16] Villegas-Aguilar M.d.C., Fernández-Ochoa Á., Cádiz-Gurrea M.d.l.L., Pimentel-Moral S., Lozano-Sánchez J., Arráez-Román D., Segura-Carretero A. (2020). Pleiotropic biological effects of dietary phenolic compounds and their metabolites on energy metabolism, inflammation and aging. Molecules.

[17] Peighambardoust S.H., Karami Z., Pateiro M., Lorenzo J.M. (2021). A review on health-promoting, biological, and functional aspects of bioactive peptides in food applications. Biomolecules.

[18] Aguilar-Toalá J., Quintanar-Guerrero D., Liceaga A., Zambrano-Zaragoza M. (2022). Encapsulation of bioactive peptides: A strategy to improve the stability, protect the nutraceutical bioactivity and support their food applications. RSC Adv..

[19] Yu J., Chen G., Jin Y., Zhang M., Wu T. (2025). Research Progress of Bioactive Peptides in Improving Type II Diabetes. Foods.

[20] Chelliah R., Wei S., Daliri E.B.-M., Elahi F., Yeon S.-J., Tyagi A., Liu S., Madar I.H., Sultan G., Oh D.-H. (2021). The role of bioactive peptides in diabetes and obesity. Foods.

[21] Manzanares P., Gandía M., Garrigues S., Marcos J.F. (2019). Improving health-promoting effects of food-derived bioactive peptides through rational design and oral delivery strategies. Nutrients.

[22] Qiao Q., Chen L., Li X., Lu X., Xu Q. (2021). Roles of dietary bioactive peptides in redox balance and metabolic disorders. Oxidative Med. Cell. Longev..

[23] Hill C., Guarner F., Reid G., Gibson G.R., Merenstein D.J., Pot B., Morelli L., Canani R.B., Flint H.J., Salminen S. (2014). Expert consensus document: The International Scientific Association for Probiotics and Prebiotics consensus statement on the scope and appropriate use of the term probiotic. Nat. Rev. Gastroenterol. Hepatol..

[24] Dahiya D., Nigam P. (2022). Probiotics, Prebiotics, Synbiotics, and Fermented Foods as potential biotics in Nutrition Improving Health via Microbiome-Gut-Brain Axis. Fermentation.

[25] Plessas S., Bekatorou A., Gallanagh J., Nigam P., Koutinas A.A., Psarianos C. (2008). Evolution of aroma volatiles during storage of sourdough breads made by mixed cultures of *Kluyveromyces marxianus* and *Lactobacillus delbrueckii* ssp *bulgaricus* or *Lactobacillus helveticus*. Food Chem..

[26] Plessas S., Fisher A., Koureta K., Psarianos C., Nigam P., Koutinas A.A. (2008). Application of *Kluyveromyces marxianus*, *Lactobacillus delbrueckii* ssp *bulgaricus* and *L. helveticus* for sourdough bread making. Food Chem..

[27] Dahiya D., Nigam P.S. (2022). The gut microbiota influenced by the intake of probiotics and functional foods with prebiotics can sustain wellness and alleviate certain ailments like gut-inflammation and colon-cancer. Microorganisms.

[28] Hijová E. (2024). Postbiotics as metabolites and their biotherapeutic potential. Int. J. Mol. Sci..

[29] Dahiya D., Nigam P.S. (2023). Therapeutic and Dietary Support for Gastrointestinal Tract Using Kefir as a Nutraceutical Beverage: Dairy-Milk-Based or Plant-Sourced Kefir Probiotic Products for Vegan and Lactose-Intolerant Populations. Fermentation.

[30] Ferris M.M., Subitoni Antonio L., Al-Sadi R. (2025). Probiotics and the intestinal tight junction barrier function. Front. Cell Dev. Biol..

[31] Dahiya D., Terpou A., Nigam P.S. (2025). Strategic Restoration of Sustainability in Gut Microbiome Diversity Through Synbiotic Food, Functional Beverages or Commercial Probiotic Formulations: Achieving Sustainable Development Goals 03, 09 and 12. SCI Sustain..

[32] Terpou A., Dahiya D., Nigam P.S. (2025). Evolving interplay between fermented food microbiota and gut microenvironment—Strategic pathways to improve human health. Foods.

[33] Combs G.F., McClung J.P. (2017). The Vitamins: Fundamental Aspects in Nutrition and Health.

[34] Tardy A.L., Pouteau E., Marquez D., Yilmaz C., Scholey A. (2020). Vitamins and Minerals for Energy, Fatigue and Cognition: A Narrative Review of the Biochemical and Clinical Evidence. Nutrients.

[35] Reboul E. (2013). Absorption of vitamin A and carotenoids by the enterocyte: Focus on transport proteins. Nutrients.

[36] McClements D.J., Li F., Xiao H. (2015). The nutraceutical bioavailability classification scheme: Classifying nutraceuticals according to factors limiting their oral bioavailability. Annu. Rev. Food Sci. Technol..

[37] Traber M.G., Stevens J.F. (2011). Vitamins C and E: Beneficial effects from a mechanistic perspective. Free Radic. Biol. Med..

[38] Mora J.R., Iwata M., von Andrian U.H. (2008). Vitamin effects on the immune system: Vitamins A and D take centre stage. Nat. Rev. Immunol..

[39] Terpou A., Nigam P., Bosnea L., Kanellaki M. (2018). Evaluation of Chios mastic gum as antimicrobial agent and matrix forming material targeting probiotic cell encapsulation for functional fermented milk production. LWT Food Sci. Technol..

[40] Bosnea L., Moschakis T., Nigam P., Biliaderis C.G. (2017). Growth adaptation of probiotics in biopolymer-based coacervate structures to enhance cell viability. LWT Food Sci. Technol..

[41] Vasiliki S., Terpou A., Bosnea L., Kanellaki M., Nigam P. (2018). Entrapment of *Lactobacillus casei* ATCC393 in the viscus matrix of *Pistacia terebinthus* resin for functional myzithra cheese manufacture. LWT Food Sci. Technol..

[42] Terpou A., Bekatorou A., Kanellaki M., Koutinas A.A., Nigam P. (2017). Enhanced probiotic viability and aromatic profile of yogurts produced using wheat bran (*Triticum aestivum*) as cell immobilization carrier. Process Biochem..

[43] Akbarian M., Khani A., Eghbalpour S., Uversky V.N. (2022). Bioactive peptides: Synthesis, sources, applications, and proposed mechanisms of action. Int. J. Mol. Sci..

[44] Kandyliari A., Potsaki P., Bousdouni P., Kaloteraki C., Christofilea M., Almpounioti K., Moutsou A., Fasoulis C.K., Polychronis L.V., Gkalpinos V.K. (2023). Development of dairy products fortified with plant extracts: Antioxidant and phenolic content characterization. Antioxidants.

[45] Wong C.L., Givens D.I., Turpeinen A.M., Liu X., Guo J. (2025). Is vitamin D fortification of dairy products effective for improving vitamin D status? A systematic review and meta-analysis of randomised controlled trials. Nutrients.

[46] Korhonen H., Pihlanto A. (2006). Bioactive peptides: Production and functionality. Int. Dairy J..

[47] Plessas S., Trantallidi M., Bekatorou A., Kanellaki M., Nigam P., Koutinas A.A. (2007). Immobilization of kefir and *Lactobacillus casei* on brewery spent grains for use in sourdough wheat bread making. Food Chem..

[48] Mandache M.B., Vijan L.E., Cosmulescu S. (2025). Insight into Bioactive Compounds and Antioxidant Activity of Bakery Products Fortified with Fruit Pomace. Foods.

[49] Dahiya D., Nigam P.S. (2023). Inclusion of Dietary-Fibers in Nutrition Provides Prebiotic Substrates to Probiotics for the Synthesis of Beneficial Metabolites SCFA to Sustain Gut Health Minimizing Risk of IBS, IBD, CRC. Recent Prog. Nutr..

[50] Morán J., Kilasoniya A. (2024). Integration of Postbiotics in Food Products through Attenuated Probiotics: A Case Study with Lactic Acid Bacteria in Bread. Foods.

[51] Almada-Érix C.N., Almada C.N., Pedrosa G.T.S., Biachi J.P., Bonatto M.S., Schmiele M., Nabeshima E.H., Clerici M.T.P., Magnani M., Sant’Ana A.S. (2022). Bread as probiotic carriers: Resistance of Bacillus coagulans GBI-30 6086 spores through processing steps. Food Res. Int..

[52] Arslan-Tontul S., Erbas M., Gorgulu A. (2019). The use of probiotic-loaded single-and double-layered microcapsules in cake production. Probiotics Antimicrob. Proteins.

[53] Kaim U., Goluch Z.S. (2023). Health benefits of bread fortification: A systematic review of clinical trials according to the PRISMA statement. Nutrients.

[54] Dahiya D., Nigam P. (2023). Use of Characterized Microorganisms in Fermentation of Non-Dairy-Based Substrates to Produce Probiotic Food for Gut-Health and Nutrition. Fermentation.

[55] Miles E.A., Calder P.C. (2021). Effects of citrus fruit juices and their bioactive components on inflammation and immunity: A narrative review. Front. Immunol..

[56] Artés-Hernández F., Castillejo N., Martínez-Zamora L., Martínez-Hernández G.B. (2021). Phytochemical fortification in fruit and vegetable beverages with green technologies. Foods.

[57] Ndovie P., Nkhata S.G., Jali N., Chisapo G., Sanuka M., Saka L., Kammwamba K., Namaumbo S., Munthali J. (2025). Microorganisms as Nutrient Factories: Harnessing Bio-fermentation for Food Fortification. Sustainable Food Fortification: Biobased Approaches and Strategies.

[58] Gunawardena S., Nadeeshani H., Amarasinghe V., Liyanage R. (2024). Bioactive properties and therapeutic aspects of fermented vegetables: A review. Food Prod. Process. Nutr..

[59] Hidalgo-Fuentes B., de Jesús-José E., Cabrera-Hidalgo A.d.J., Sandoval-Castilla O., Espinosa-Solares T., González-Reza R.M., Zambrano-Zaragoza M.L., Liceaga A.M., Aguilar-Toalá J.E. (2024). Plant-based fermented beverages: Nutritional composition, sensory properties, and health benefits. Foods.

[60] Görgüç A., Gençdağ E., Yılmaz F.M. (2020). Bioactive peptides derived from plant origin by-products: Biological activities and techno-functional utilizations in food developments–A review. Food Res. Int..

[61] Sosalagere C., Kehinde B.A., Sharma P. (2022). Isolation and functionalities of bioactive peptides from fruits and vegetables: A reviews. Food Chem..

[62] Baker M.T., Lu P., Parrella J.A., Leggette H.R. (2022). Consumer acceptance toward functional foods: A scoping review. Int. J. Environ. Res. Public Health.

[63] Yuan X., Zhong M., Huang X., Hussain Z., Ren M., Xie X. (2024). Industrial production of functional foods for human health and sustainability. Foods.

[64] Kalita P., Bhattacharyya J., Dutta P.P., Chakrabarti S., Ahmed A.B., Pachuau L. (2025). Valorization of polyphenolic compounds via encapsulation: A review. J. Food Meas. Charact..

[65] Sun-Waterhouse D. (2011). The development of fruit-based functional foods targeting the health and wellness market: A review. Int. J. Food Sci. Technol..

[66] Lobine D., Rengasamy K.R., Mahomoodally M.F. (2022). Functional foods and bioactive ingredients harnessed from the ocean: Current status and future perspectives. Crit. Rev. Food Sci. Nutr..

[67] Bińkowska W., Szpicer A., Stelmasiak A., Wojtasik-Kalinowska I., Półtorak A. (2024). Microencapsulation of Polyphenols and Their Application in Food Technology. Appl. Sci..

[68] Szpicer A., Bińkowska W., Stelmasiak A., Wojtasik-Kalinowska I., Czajkowska A., Mierzejewska S., Domiszewski Z., Rydzkowski T., Piepiórka-Stepuk J., Półtorak A. (2025). Innovative Microencapsulation Techniques of Bioactive Compounds: Impact on Physicochemical and Sensory Properties of Food Products and Industrial Applications. Appl. Sci..

[69] Baruroh D., Suselo Y.H., Kusumawati R., Indarto D. (2025). Effect of Freeze-Drying, Spray-Drying, and Foam-Mat-Drying Encapsulation Techniques on Vitamin C Level in Fruit Powder: A Scoping Review. J. Health Nutr. Res..

[70] Martinović J., Ambrus R., Planinić M., Šelo G., Klarić A.-M., Perković G., Bucić-Kojić A. (2024). Microencapsulation of grape pomace extracts with alginate-based coatings by freeze-drying: Release kinetics and in vitro bioaccessibility assessment of phenolic compounds. Gels.

[71] Ștefănescu B.E., Nemes S.-A., Teleky B.-E., Călinoiu L.F., Mitrea L., Martău G.A., Szabo K., Mihai M., Vodnar D.C., Crișan G. (2022). Microencapsulation and bioaccessibility of phenolic compounds of Vaccinium leaf extracts. Antioxidants.

[72] Rezagholizade-Shirvan A., Soltani M., Shokri S., Radfar R., Arab M., Shamloo E. (2024). Bioactive compound encapsulation: Characteristics, applications in food systems, and implications for human health. Food Chem. X.

[73] Browning L.W., Wang H., Taylor J.W., Wilde P., Rodriguez-Garcia M., Holland L.A.M., Knowles T.P. (2025). Digestibility and enteric release achieved with microencapsulates made from emulsion-templated plant proteins. Sustain. Food Technol..

[74] Vallejo-Castillo V., Rodríguez-Stouvenel A., Martínez R., Bernal C. (2020). Development of alginate-pectin microcapsules by the extrusion for encapsulation and controlled release of polyphenols from papaya (*Carica papaya* L.). J. Food Biochem..

[75] Liu S., Fang Z., Ng K. (2022). Incorporating inulin and chitosan in alginate-based microspheres for targeted delivery and release of quercetin to colon. Food Res. Int..

[76] Chehreara A., Tabandeh F., Otadi M., Alihosseini A., Partovinia A. (2022). Enhanced survival of *Lacticaseibacillus rhamnosus* in simulated gastrointestinal conditions using layer-by-layer encapsulation. Biotechnol. Lett..

[77] Saiz-Gonzalo G., Arroyo-Moreno S., McSweeney S., Bleiel S.B. (2025). Pea protein microencapsulation improves probiotic survival during gastrointestinal digestion. Int. J. Food Sci. Technol..

[78] Liu L., Fishman M.L., Kost J., Hicks K.B. (2003). Pectin-based systems for colon-specific drug delivery via oral route. Biomaterials.

[79] Mehta N., Kumar P., Verma A.K., Umaraw P., Kumar Y., Malav O.P., Sazili A.Q., Domínguez R., Lorenzo J.M. (2022). Microencapsulation as a noble technique for the application of bioactive compounds in the food industry: A comprehensive review. Appl. Sci..

[80] da Silva Júnior M.E., Araújo M.V.R.L., Martins A.C.S., dos Santos Lima M., Da Silva F.L.H., Converti A., Maciel M.I.S. (2023). Microencapsulation by spray-drying and freeze-drying of extract of phenolic compounds obtained from ciriguela peel. Sci. Rep..

[81] Abdel-Aty A.M., Barakat A.Z., Bassuiny R.I., Mohamed S.A. (2024). Chia gum-gelatin-based encapsulation of chia sprouts phenolic compounds enhanced storage stability, bioavailability, antioxidant, antidiabetic, and antibacterial properties. Sci. Rep..

[82] Duque-Soto C., Leyva-Jiménez F.J., Quirantes-Piné R., López-Bascón M.A., Lozano-Sánchez J., Borrás-Linares I. (2023). Evaluation of Olive Leaf Phenolic Compounds’ Gastrointestinal Stability Based on Co-Administration and Microencapsulation with Non-Digestible Carbohydrates. Nutrients.

[83] Mueller D., Jung K., Winter M., Rogoll D., Melcher R., Kulozik U., Schwarz K., Richling E. (2018). Encapsulation of anthocyanins from bilberries—Effects on bioavailability and intestinal accessibility in humans. Food Chem..

[84] Gruskiene R., Bockuviene A., Sereikaite J. (2021). Microencapsulation of bioactive ingredients for their delivery into fermented milk products: A review. Molecules.

[85] Zhao X., Ai Y., Hu Y., Wang Y., Zhao L., Yang D., Chen F., Wu X., Li Y., Liao X. (2020). Masking the perceived astringency of proanthocyanidins in beverages using oxidized starch hydrogel microencapsulation. Foods.

[86] Grassia M., Messia M., Marconi E., Demirkol Ȫ.Ş., Erdoğdu F., Sarghini F., Cinquanta L., Corona O., Planeta D. (2021). Microencapsulation of phenolic extracts from cocoa shells to enrich chocolate bars. Plant Foods Hum. Nutr..

[87] Motilva M.-J., Macià A., Romero M.-P., Rubió L., Mercader M., González-Ferrero C. (2016). Human bioavailability and metabolism of phenolic compounds from red wine enriched with free or nano-encapsulated phenolic extract. J. Funct. Foods.

[88] Wang M., Li S., Chen Z., Zhu J., Hao W., Jia G., Chen W., Zheng Y., Qu W., Liu Y. (2021). Safety assessment of nanoparticles in food: Current status and prospective. Nano Today.

